# The functional form of the association between K-12 student performance and household income in U.S. school districts

**DOI:** 10.1371/journal.pone.0329296

**Published:** 2025-09-10

**Authors:** Maria N. Wodarz, Natalia L. Komarova, Timmy Ma

**Affiliations:** 1 University High School, Irvine, California, United States of America; 2 Department of Mathematics, University of California, San Diego, La Jolla, California, United States of America; 3 Department of Mathematics, Xavier University of Louisiana, New Orleans, Louisiana, United States of America; University College London, UNITED KINGDOM OF GREAT BRITAIN AND NORTHERN IRELAND

## Abstract

It is well-known that income can correlate with the academic performance of K-12 students in the United States (U.S.). However, the mathematical relationship between income and K-12 performance, and how it varies across states, remains poorly understood. To help fill this gap, this study examines the relationship between K-12 student performance scores (defined as the percentage of students meeting or exceeding grade-level expectations) and median household income, across more than 12,200 public school districts in 42 US states. The study focuses on performance in English Language Arts (ELA) and Mathematics in 3rd and 8th grades during the 2018-2019 school year. A number of different mathematical functions are explored to quantitatively characterize this relationship, and the best fitting functions are determined statistically. It was found that in about half of the states, the proficiency rate increases linearly with the median household income, while in the rest of the states the increase is characterized by a saturating function. Further, the results reveal that less affluent states exhibit a steeper increase in performance with income compared to wealthier states. Additionally, grade-level and subject comparisons highlight disparities, including a pronounced decline in math performance from 3rd to 8th grade in most districts. These findings underscore the correlations between socioeconomic factors and educational outcomes and the variations between subjects, grade levels, as well as locations. By expanding our understanding of these relationships, this research offers potentially useful mathematical methodologies for developing evidence-based, quantitative approaches to studying educational equity.

## Introduction

Educational performance in K-12 settings has long been recognized as a critical determinant of economic mobility and societal progress. Numerous studies have explored the complex interplay of factors influencing student achievement, with socioeconomic status emerging as one of the most prominent predictors, see e.g. [1, 2] for early reports on the strong correlation between family socioeconomic status and educational outcomes. Since then, many authors have discussed the causes and mechanisms of this relationship, see e.g. [3–6].

The strong link between income and educational performance is often attributed to wealthier districts’ ability to provide superior facilities, technology, and extracurricular opportunities [7–9]. Studies have also pointed to the role of parental involvement and home learning environments, which tend to be richer in high-income households, as key contributors to the achievement gap [10, 11]. Additionally, disparities in teacher-to-student ratios and teacher experience further contribute to this phenomenon [12]. Disparities rooted in access to resources, teacher quality, and infrastructure have also been highlighted [13–16]. The relationship between income inequality and student achievement has been studied by [17, 18], who found that states with higher income inequality had lower average math scores. A number of other studies have also suggested that math performance may be more sensitive to socioeconomic disparities than literacy [19, 20]. While the correlations between income and performance are well-documented, the quantitative nature of this relationship and its variations across states, subjects, and grade levels remain less understood.

Different methodologies have been implemented to measure the income achievement gap, and different data sources have been used. [8] used data from several nationally representative samples of students, including the National Center for Education Statistics (NCES), the Long Term Trend National Assessment of Educational Progress (NAEP-LTT), and the Main National Assessment of Educational Progress (NAEP) studies. The authors defined the “90-10 income achievement gap” as the difference in average scores between students whose family incomes were in the 90th and 10th percentile of the national family income distribution. Paper [21] used data from four repeated studies, including NAEP-LTT, NAEP, and parts of international assessments. Although these studies did not include information on family income, the authors constructed indices of family socioeconomic status (SES) based on background questionnaires that contained information about parents’ education and educational possessions at home. As a measure of achievement gap, the authors estimated the average test score difference between students in the top and bottom quartile of the SES measures. Paper [22] used data from the NAEP assessments. Similar to paper [21], information on family income was not included, thus the authors implemented a multiple-step estimation approach that used information on the income distribution among families living near sampled schools to construct estimates of the achievement gap. These studies, as well as [23], focused on the question of whether or not the income-achievement gap in the U.S. has widened or narrowed throughout the last few decades.

In the present study we do not consider temporal trends in the achievement gap, but instead focus on quantifying correlations between public school performance and income. Specifically, we use performance data collected at the level of U.S. public school districts, coupled with the information on median household income from each district. The dataset we created contains more than 12,200 public unified school districts from 42 U.S. states. We examine the functional dependence between median household income and K-12 student performance in English and Mathematics for two grade levels (3rd and 8th). We test a number of mathematical relationships, and identify statistically the strongest mathematical model that relates performance to income. These mathematical relationships enable us to quantify observations that vary across different states, compare trends between subjects, and analyze grade-level differences. By providing a comprehensive state-by-state analysis, this work contributes to a deeper understanding of the correlations between income and school performance and resulting educational disparities.

## Materials and methods

We have collected and analyzed the unified-school-district-level education data from 42 U.S. states, see [24]. S1 Appendix, Sect A provides a description of the data sources and other details of the data collection process. The information collected for more than 12,200 public unified school districts across the states was what we will call the “performance score". It is defined as the percentage of students, in each school district, who performed at or above their grade level in their respective states’ assessment exams. This score was obtained for the school year 2018-2019. It is important to note that each state has its independent achievement indicator of what they consider to be “meeting the state standards" or “exceeding the state standards" for each grade level, and these indicators can vary from state to state.

Ethical approval was not needed for this work, because the study used publicly available data from official school district sites, as explained in detail in S1 Appendix, Sect A. These data are aggregate (test result percentages over the whole school district), publicly available, and were not collected for the purposes of this study. The data were accessed by us in Fall 2024. The authors had no access to information that could identify individual participants during or after data collection.

In this study, we focus on two subjects: English Language Arts (ELA, or equivalent reading subject score if a state did not specify ELA in their data) and Mathematics (Math). We collected the data for two grade levels, 3rd and 8th grade. Therefore, in this study, we consider four subject-grade combinations: ELA 3, Math 3, ELA 8, and Math 8. Below we perform pairwise comparisons by subject within the same grade level and by grade level within the same subject.

In addition to the educational information, we included information on each school district’s median household income, see [24], as well as the median household income for all the U.S. states (see S1 Appendix, Sect A for details).

We fitted the performance score versus median household income data for each state at the district level. We used several methods to fit the data:

1. The usual least square method, using the data for each district. This standard method may have a shortcoming, in which intermediate incomes are much more represented in the datasets compared to very low and very high incomes, so the best fitting function may not correctly describe these ranges.
2. The binned fitting method where the income was split into intervals ($5,000), the districts grouped by income, and for each group, the mean income and the mean educational score were calculated. Then the least square fitting is performed for these mean values. This method may be preferred to the previous one because it increases the relative contribution of very low- and very high-income groups.
3. Same as before, the data are split into bins by income, and then weighted fitting is performed for the mean values, where the weight is the inverse standard error of the mean for each of the bins.

We repeated the same procedures for the performance score versus per capita income. The results were very similar, so in the rest of the paper we focus on the performance score versus median household income data.

Several different functions were used to fit the data:

- One-parametric functions:
  $$y=ax;\;y=1-e^{-ax};\;y=1-a/x;\;y=1-a/x^{2}.$$
- Two-parametric functions:
  $$y=bx+c;\;y=b-e^{-ax};\;y=1-be^{-ax};\;y=b-a/x;\;y=b-a/x^{2};\;y=b+a\ln(x).$$
- Three-parametric functions:
  $$y=b-ce^{-ax};\;y=b/(1+e^{-a(x-c)}).$$

(Note that the parameters *a*, *b*, *c* in the above functions are only meant to indicate “an unknown parameter", and do not denote the same value across different functions). Using the Bayesian information criterion (BIC), we selected the two best models, which were the two-parametric linear function and the one-parametric exponential function. The pairwise comparison between these two functions was also performed by using the BIC. These results were very similar for all three methods of fitting that we used. In the next section, we present results for the third fitting method (the weighted least squared fitting).

## Results

In order to investigate the dependence of K-12 student performance on the state assessments with median household income in the U.S., we collected school-district level information from 42 U.S. states. For each of the school districts in those states, we focused on the ELA and the Mathematics performance of public school students in 3rd and 8th grade from the school year 2018-2019. For each of the four subject-grade combinations, we define as the “performance score" the percentage of students who performed at or above their grade level on their state assessment test, as determined by the respective state’s Department of Education.

**Fig 1:**
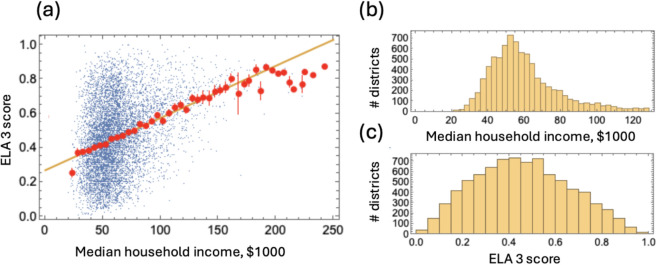
All the district data for ELA 3 combined. (a) The score versus income dependence. Each blue dot represents a district and shows the ELA 3 score versus the district’s median household income. The yellow line is the best-fitting linear function (y=0.27+0.003x), and the red dots and bars represent the mean and standard error of the scores in districts binned by income with a $5,000 step. (b) The mean household income of the districts represented as a histogram. (c) The ELA 3 score of the districts represented as a histogram.

Fig 1 shows all the data for ELA 3 scores plotted together. In panel (a) each district is represented as a blue dot. Districts are then combined together by income (with a step size of $5,000) and the mean and standard error of the score are plotted by red dots. We note the remarkable linear increase of the mean score as a function of the income over a very large span of incomes (from about $30,000 to about $170,000). The best linear fit of all the data is shown by the yellow line which aligns almost perfectly with the means of the binned scores. Panels (b) and (c) show the overall distributions of incomes and ELA scores over all the districts.

The trend line (with the slope of *b* = 0.0030 per $1,000) tells us that an increase of the household income by $10,000 results on average in a 3% increase in the number of students that perform at or above the grade level (in ELA, 3rd grade). The results for other subject-grade combinations are similar: for Math 3 we have *b* = 0.0030, for ELA 8 it is *b* = 0.0035 and for Math 8 it is *b* = 0.0027.

While these summary data are interesting, we must note that both performance scores and income values may differ from state to state. We therefore proceed to analyze the data on a state-by-state level.

### The best fitting performance versus income function

**Fig 2:**
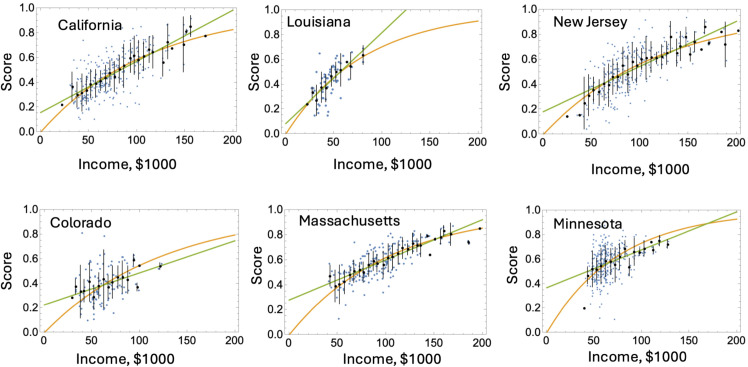
Typical examples of performance score versus median household income scatter-plots (blue dots) for ELA 3 in six U.S. states. Each blue dot represents a school district. The vertical bars represent the means and standard deviations of the scores in $5,000 bins by income. The continuous yellow and green lines represent the best fit by the functions *f*_1_(*x*) and *f*_2_(*x*), respectively. Top row: the fit by *f*_1_(*x*) is better. Bottom row: the fit by *f*_2_(*x*) is better. See S1 Appendix, Sect B, S1, S2, S3 Figs for more state plots.

Fig 2 shows several typical examples of performance scores versus median household income scatter-plots (blue dots) for the public school districts. Here, the ELA 3rd grade performance scores are presented. Graphs for the other three subject-grade combinations are qualitatively similar. Presented in these graphs are also the means and standard deviations of the performance scores by income bins, see the vertical bars in the plots.

**Fig 3:**
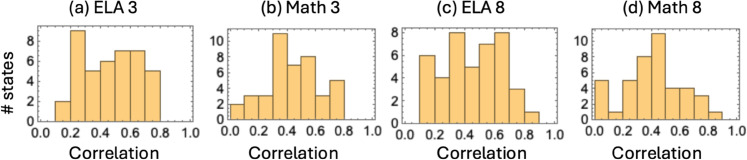
Histograms of the Pearson correlation coefficients between scores and the income for the four subject-grade combinations, across all the states.

We observe a trend where districts with higher income have higher performance scores than those with lower income. Fig 3 shows histograms of the Pearson correlation coefficients between the performance score and the income for all the states. It is positive for all the states and all the subject-grade combinations, although the correlation strength varies.

To determine the nature of the relationship between performance scores and median household income, we fitted a number of one, two-, and three-parametric functions to the sets of performance scores versus income for each state (see Materials and Methods for details; 12 functions were used altogether). All the functions were increasing functions of income. The rate of increase was constant in the two linear functions, and it decreased with income in the other 10 functions. Of these, 9 functions were saturating (that is, they had a horizontal asymptote), and one was unbounded (namely, the function *y* = *b*  +  aln(x)). The linear function and the function y=b+aln(x) have been previously used in the literature to represent the dependence of educational outcomes on income (see [21, 23]).

**Fig 4:**
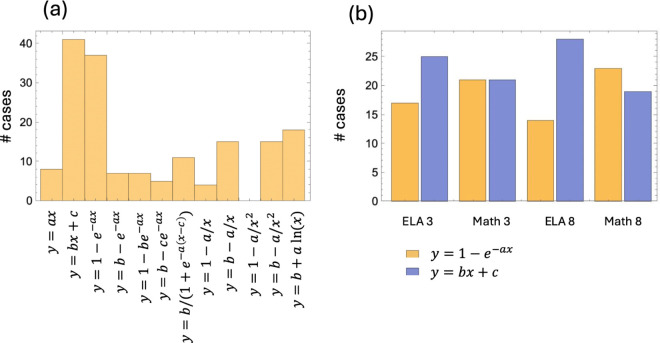
Model comparison for score versus income dependency. (a) Twelve different functions were used to fit the score versus income data for each of the states and each subject/grade combination. The quality of the model was evaluated by using the BIC. The bar graphs show in how many cases each of the models was chosen to be the best description of the data. (b) Comparing models $y=1\;-\;e^{-ax}$ and y=bx+c by using the BIC. The bar graphs show in how many cases each of the models was chosen to be the best description of the data, for each subject/grade combination. Altogether, the nonlinear model was selected in 90 out of 168 cases.

Fitting was performed by using the usual least squares procedure, and Bayesian information criterion (BIC) was applied to determine the best fit. Fig 4(a) presents the results of the model comparison for all 12 functions. Two of the functions demonstrated significantly better performance across all states, grade levels, and subjects: a one-parametric saturating function,

$$y=f_{1}(x)=1-\exp(-ax),$$ (1)

and a two-parametric linear function,

$$y=f_{2}(x)=bx+c,$$ (2)

where *x* denotes the income (in $1000), *y* denotes the performance score, and a,b, and *c* are the parameters that we fitted to the data. Analysis of the parameters *a* and *b* are discussed below.

When comparing functions *f*_1_(*x*) and *f*_2_(*x*) with each other, it turned out that in about half of the states, the linear function (Eq 2) is the best model by the BIC, while the nonlinear model with saturation (Eq 1) is chosen to be the best in the other half of the states, see Fig 4(b).

In Fig 2, both fits are shown, with the yellow line representing the saturating function (1) and the green line representing the linear function (2). The three states in the top row (California, Louisiana, and New Jersey) were chosen among the states where function (1) has a lower BIC score (and thus is considered a stronger model). The states in the bottom row (Colorado, Massachusetts, and Minnesota) are from the group of states where the linear function is a better description. In S1 Appendix, Sect B we present graphs for the ELA 3 score versus median household income for all the states, together with the best fitting functions (1) and (2). We group all the states according to which function is a better fit. Data on the other subject/grade combinations look similar. For Math 8, the group of states where the nonlinear function is a better fit is somewhat larger.

From these model comparisons we can see that with the data presently available, we cannot conclude with certainty that one of the two candidate functions of score versus income is universally better than the other. While it is common sense to assume that a saturating function should be expected to describe a quantity bounded by one (the performance score is a fraction), this deviation from linearity is not observed for a number of states, possibly because of the scarcity of data for higher-income districts and the lack of data on private school student performance.

For relevant ranges of median incomes the two functions have a similar shape (see e.g. S1 Appendix, Sect B, S1, S2, S3 Figs). In what follows, we will use parameters aandb in functions (1) and (2), respectively to characterize the rate of the score change as a function of the median household income.

### Comparison across states

**Fig 5:**
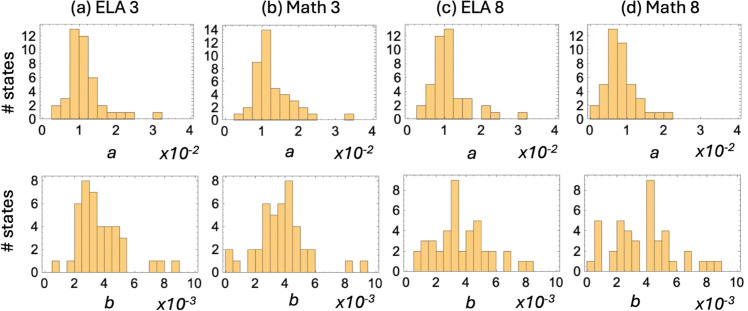
Parameters of the score versus income functions for different states. Top row: Histograms of the best fitting rate of change, *a*, in the saturating fit *f*_1_(*x*) (Eq 1). The horizontal axis represents the values of amultipliedby100. Bottom row: Histograms of the best fitting rate of change, *b*, in the linear fit, *f*_2_(*x*) (Eq 2). The horizontal axis represents the values of bmultipliedby1000.

Fig 5 presents histograms of the best-fitting values of the slope coefficient in both winning models in the four subject-grade combinations, for all the states. We can see that the *a*-values, which are the slope parameters in the saturating function (the top row) cluster about their means given by ⟨a⟩=0.01 for ELA 3, Math 3, and ELA 8 (with *p*>0.1 by T-test), while for Math 8 the values are somewhat lower, with ⟨a⟩=0.008(p<0.05) by T-test when comparing with the other subject-grade combinations). Similar trends are seen when fitting with the linear function. The *b*-values (the slope parameters in the linear function shown in the bottom row of Fig 5) cluster about their means given by ⟨b⟩=0.0036 for all the subject/grade combinations (with *p*>0.1 by T-test).

**Fig 6:**
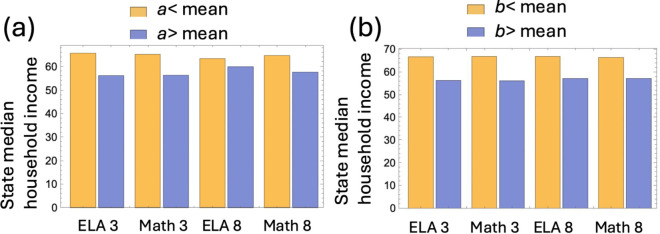
Characteristics of states with lower (yellow) and higher (blue) values of the rate of change. (a) Median household income (in $1,000) for states with lower than and higher than the average value of *a*. The difference of the means between low-*a* and high-*a* groups is significant (*p*<0.05 by T-test) except for the median household income for ELA 8. (b) Median household income (in $1,000) for states with lower than and higher than the average value of *b*. The difference of the means between low-*b* and high–*b* groups is significant (p<0.05 by T-test) for all cases.

In order to investigate the variation of the rate of change, *a*, among the states, we categorized the states into two groups: those whose estimated *a*–value is below the overall mean ⟨a⟩ (yellow bars in Fig 6(a)), and those whose *a*–value is above the mean ⟨a⟩ (blue bars in Fig 6(a)). Then we compared the median household income of the states in these two groups. The results were very consistent. For all the subject-grade combinations, the states with lower values of the rate of change, *a*, were characterized by a higher (on average) median household income (represented by the yellow bars). The states with higher values of the rate of change, *a*, were characterized by a lower (on average) median household income (represented by the blue bar). A similar pattern was observed when we compared the rate constants obtained by fitting the linear function, see Fig 6(b).

This result can be interpreted as follows: a higher household income is associated with a lower rate of change in student performance with income. This means that a fixed increase in income amounts to a larger performance score increase in less wealthy locations.

Using the fitted parameters (see Fig 5), one can estimate the expected difference in the performance score as the income increases. For the typical value of the slope $b\approx 3.6\;\times \;10^{-3}$ for function (2), we can see that a $10,000 difference in the median household income will increase a district’s performance score by about 3.6%.

For those states where the saturating function, Eq 1, represents the score versus income dependence, we observe a decrease in the effective rate of score change with income. In those cases, the score improvement per $10,000 is higher for low-income districts compared to high-income districts. For example, in California (ELA 3), parameter *a* = 0.009, we expect a 6.0% increase in score per a $10,000 household income increase for school districts with a $40,000 median household income. This should be compared with only a 1.4% score increase if the median household income is $200,000.

### Comparisons across subjects and grade levels

**Fig 7:**
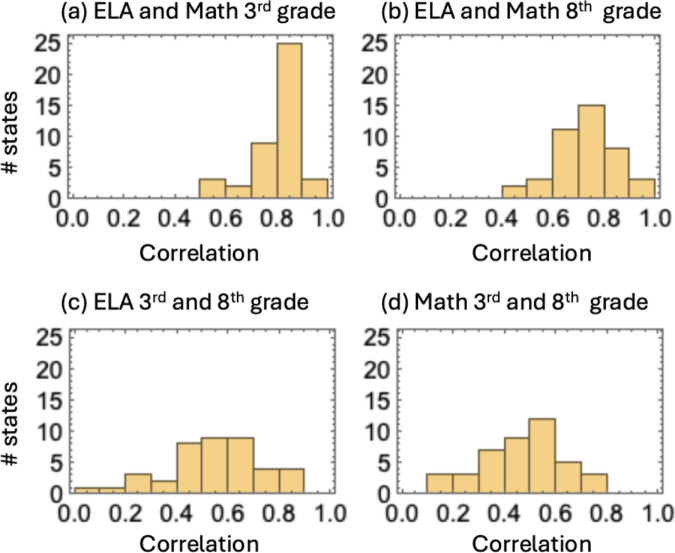
Pearson correlations between different subjects (panels (a) and (b)) and different grade levels (panels (c) and (d)) for all the states.

To investigate differences between ELA and Math performance, we calculated the Pearson correlation coefficients (PCC) between those subject’s scores for each state. Fig 7(a) shows a histogram of PCC for students in the 3rd grade and panel (b) shows the same quantities for the 8th grade. We observe a strong correlation in all the states in the 3rd grade, with more than three-fourths of all the states having PCC >0.75. The correlation weakens somewhat in the 8th grade but remains significant with more than half of the states with PCC >0.75.

Turning to correlations between the two grade levels, Fig 7(c) shows a histogram of PCC between ELA 3rd- and 8th-grade performance, while panel (d) shows the same quantities for Math. In all the states, PCC is positive, but clearly, the values are weaker compared to, for example, panel (a). To investigate this further, we looked into temporal trends in performance within each of the subjects.

**Fig 8:**
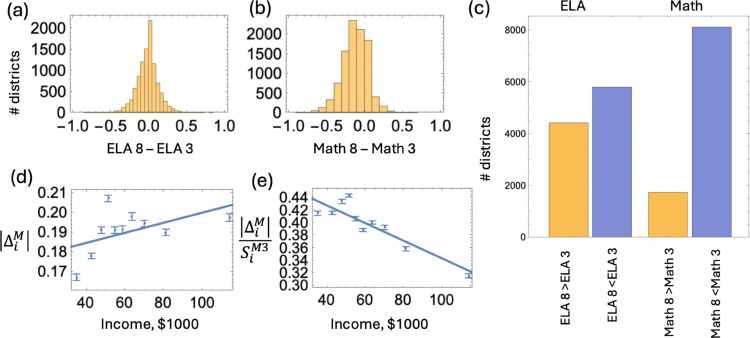
Dependence of performance on the grade level. (a) The difference in the ELA score between the 8th and the 3rd grade, $\Delta_{i}^{E}=S_{i}^{E8}-S_{i}^{E3}$, by district, represented as a histogram (negative values represent a worse performance in the 8th compared to the 3rd grade). (b) The same for Math: $\Delta_{i}^{M}=S_{i}^{M8}-S_{i}^{M3}$. (c) The number of districts with an increase (yellow) and decrease (blue) in performance between 3rd and 8th grade. ELA results are shown on the left and Math results on the right. (d) For districts with $S_{i}^{M3}>S_{i}^{M8}$, the vertical bars show the mean and the standard error of the drop in Math performance, as a function on income, by 10 quantiles. The straight line shows the linear regression. (e) The same for the relative drop in Math performance.

For district *i*, denote by $S_{i}^{E3}$, $S_{i}^{M3}$, $S_{i}^{E8}$, and $S_{i}^{M8}$ the performance score in ELA 3, Math 3, ELA 8, and Math 8, respectively. The quantity $\Delta_{i}^{E}=S_{i}^{E8}$ − $S_{i}^{E3}$ is the difference between the ELA score in the 8th grade and that in the 3rd grade in district *i*. In Fig 8(a), we show a histogram of $\Delta_{i}^{E}$ for all the school districts in all the states. If $\Delta_{i}^{E}>0$ then for district *i*, the percentage of students performing at or above the grade level increased from 3rd to 8th grade. If $\Delta_{i}^{E}<0$, then for district *i*, we have a lower performance in the 8th grade compared to the 3rd grade. We can see a roughly symmetric one-peak distribution of such score differences. This is confirmed by the bar chart in panel (c), left, where the number of districts with a decrease in performance is only slightly higher than the number of districts with an increase in performance in ELA.

When we performed the same analysis for Math (Fig 8(b)), we saw a very different result. The histogram of the performance differential is clearly asymmetric with a heavier portion in the negative values. The bar graph on the right in panel (c) shows that in a vast majority (more than 82%) of all the districts, the percentage of students performing well in math decreases from 3rd to 8th grade. We asked the question: how do performance differences between the 8th and the 3rd grade correlate with income?

As mentioned above, $\Delta_{i}^{E}$ measures the performance difference in ELA between the 8th and the 3rd grade, and $\Delta_{i}^{M}$ measures the difference in Math performance. We calculated the PCC between the performance score difference ($\Delta_{i}^{E}$ and $\Delta_{i}^{M}$) and income in the school districts; note that for this analysis we did not perform a cost-of-living adjustment. Interestingly, there is a slight positive correlation in the case of ELA (PCC = 0.08) and a slightly negative correlation in the case of Math (PCC = −0.07). To look more closely at the dynamics of Math achievement and its dependence on income, we considered the districts where Math performance drops from 3rd to 8th grade, that is, $\Delta_{i}^{M}<0$, and studied the dependence of the absolute value of the decrease, $\left|\Delta_{i}^{M}\right|$, on the income. Performing linear regression, we found that $\left|\Delta_{i}^{M}\right|$ correlates positively with income (*p*<10^−6^), see Fig 8(d). In other words, the drop in Math performance is somewhat deeper for wealthier districts. This can be explained by a significantly higher starting point ($S_{i}^{M3}$) for wealthier districts. Alternatively, this may be the result of a larger percentage of students selecting out of public schools in wealthier districts.

If, however, we consider the relative drop in performance score, $|\Delta_{i}^{M}|/S_{i}^{M3}$, the trend reverses. We find that $|\Delta_{i}^{M}|/S_{i}^{M3}$ is negatively correlated with income (*p*<10^−6^), see Fig 8(e). This means that the relative drop in mathematics performance is higher in poorer districts and lower in the wealthier districts.

## Discussion

This study investigated the functional form of the mathematical relationship between K-12 student performance and median household income across school districts in 42 U.S. states during the 2018–2019 academic year, focusing on English Language Arts (ELA) and Mathematics performance in 3rd and 8th grades, and how these functional forms might differ across states. Model selection procedures identified a one-parameter saturating (exponential) model and a two-parameter linear model as the best descriptors of the relationship between district income and the percentage of students meeting or exceeding grade-level expectations. In some states, the saturating function was selected by the BIC, while in others, a linear description was selected. We ascribe this to the scarcity of data for the higher income range, such that the saturating effect is not always statistically significant. Among the states where the exponential model yielded the best fit, significant variation among states in the parameter that characterizes this relationship was observed.

**Fig 9:**
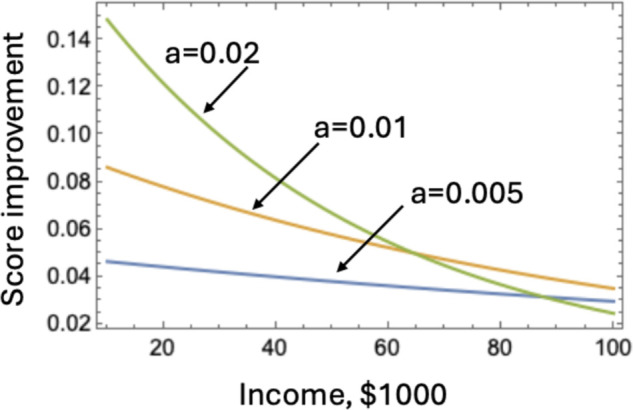
Three examples showing how the expected performance score improvement (per 10,000 added income) changes as a function of income, for the rate of change a = 0.005, a = 0.01, and a = 0.02. The function plotted is $f_{1}(x+10)-f_{1}(x)$, where *x* is the income measured in 1,000.

Consistently for all the states, a positive correlation between income and performance was found, with less affluent states associated with steeper increases in performance as income rises, compared to wealthier states. On average, the slope of the score versus median household income corresponds to about a 3.6% score increase per each $10,000 of household income added. For those states where the score versus income dependence is represented by a saturating function, the score improvement per $10,000 added is higher for low-income districts compared to high-income districts. For example, in California, a 6% increase is observed for the least wealthy, and a much lower 1.4% increase is expected for the wealthiest districts. Our mathematical analyses indicates that the rate of change, *a*, is different from state to state, and it is the parameter *a* that characterizes the differences between different income groups, as illustrated in Fig 9. For three different values of *a* (all within the range of the parameter values measured for the different states), we can see that the score improvement per $10,000 added income attains different values. For *a* = 0.005 (which is below the mean *a* that we measured, see Fig 9, top row), we can see that in a district whose median household income is low ($20, 000), scores can expect an improvement of 4.1%, and that in a district whose median household income is high ($80, 000), they will see similar levels of improvement. However, for higher values of *a*, we can observe a drastic difference of improvement between low-income and high-income districts.

The results provide quantitative evidence that socioeconomic factors influence educational outcomes differently across states, subjects, and grade levels. This work adds to prior studies of the income-achievement gap [8, 25–27] by offering a mathematical modeling approach for analyzing this relationship across diverse settings. Our methodology revealed that the steepness of income-performance curves varies significantly between states, with less affluent states showing greater sensitivity.

Furthermore, the identification of subject- and grade-specific patterns contributes to the growing literature on how socioeconomic disparities affect different areas of academic performance and evolve over time, see e.g. [28–30]. The results for math, in particular, align with prior research suggesting that math performance may be more sensitive to economic disparities than ELA performance due to its cumulative nature and dependency on consistent, high-quality instruction [31].

**Study limitations.** While this study provides a quantitative way to represent performance versus income dependency, there are a number of limitations that have to be noted when interpreting the results. The data that are used only pertain to performance scores in public school districts, and do not include information about performance in private schools, or the percentage of students that attend public versus private schools by district. Adding this information could differentially affect results in different income brackets, and also in different age-groups (3rd grade versus 8th grade).

We used proficiency rate to discuss K-12 performance, where we track the percentage of students that perform at the grade level or higher. This is different from actual achievement scores, which could provide additional information on student performance, as our proficiency rate has a binary nature and “misses" high achievement. While providing more granularity, a disadvantage of using measures like GPA however is that it may be more idiosyncratic and school-dependent, whereas proficiency rates rely on standards that are uniform within a state.

Moreover, while the analysis performed within each state (such as discovering the functional dependence of performance score on income) is valid, it becomes more difficult to compare different states with each other. Although the median income in each state is something that can be easily measured, direct comparisons may require cost-of-living adjustments, as well as other adjustments, such as accounting for the differences in the school system across states, differences in state educational standards (which define the grade level for each subject), etc.

**Interpretation and future directions.** The study highlights the strong correlation between K-12 student performance and median household income, emphasizing the amplified impact of income disparities on educational outcomes, particularly in less affluent regions. In particular, it is found that performance increase versus income is steeper in low-income districts. An important question is whether and in what way can we use these results as practical tools? Can we translate them into some sort of policy recommendations? We would like to emphasize that the present results should be interpreted only as correlations and functional dependencies, as opposed to causal relationships. Further developments have to be made in order to translate these results into specific policy recommendations.

The independent variable in this study is the median household income. While family income affects school performance, this is not something that can be manipulated directly through district and state policies. Other factors such as school spending, revenue, or resources should be included if we were to talk about achievement “returns" to investment. Further, in order to relate an increase in funding/investment to changes in school performance in a given district, detailed longitudinal studies are needed, such as e.g. [32–36]. To advance toward actionable recommendations, future research should incorporate district-level variables such as per-pupil expenditures, teacher qualifications, and programmatic investments, which would enable modeling of achievable returns on specific educational interventions, see also [37–39].

The mechanisms underlying the observed variations in income-performance relationships across states are complex and need further exploration. This could involve analyzing the role of state-specific funding models, teacher-student ratios, or other systemic factors [40, 41]. In the present study, the only variable considered was income. It is well known that other factors such as gender, race, bilingualism, etc are strongly correlated with school performance, see e.g. [42–44]. These factors should be included in the analysis as a necessary step of model refinement. Further, as already mentioned, longitudinal studies are needed to track how disparities evolve as students progress through the K-12 system, providing a deeper understanding of when and why gaps widen over time [37–39].

Our analysis uses school-district level data, where the performance is measured as a percentage of students at or above the grade level, and the income is the median household income of the district. While analysis at this scale is useful, we know that it does not include the complexity that exists at the scale of individual students or groups of students. For example, in the study of racial achievement gaps [45], it was shown that the racial achievement gap in mathematics among low-performing students has decreased between grades 3 and 8, while that among high-performing students widened. This degree of analysis would be impossible with the present dataset because of the absence of information about individual students.

In an analogy with physics or biology, studies performed at different spatial scales introduce different and complementary insights into the system. Our “scale" in this study is school districts and U.S. states. Future “multi-scale" studies may shed further light on the educational disparities, and suggest ways to improve the education system.

**Conclusion.** This study identified mathematical relationships between K-12 student performance and household income in the United States, and has shown that there is significant heterogeneity in this relationship across different states. This in turn was captured in the value of a parameter that characterizes this relationship, which can be a useful summary measure of the states’ performance versus median income dependencies. More work needs to be performed to connect this analysis with resource allocation policies, including relevant longitudinal district-level studies. By expanding our understanding of these relationships, this research lays initial mathematical groundwork for developing this type of evidence-based, quantitative approaches to studying educational inequity.

## Supporting information

S1 Appendix Additional information. Data collection information and performance versus income graphs for all the states.
(PDF)
