# Supplementary material for: The functional form of the association between K-12 student performance and household income in U.S. school districts
Source: PLoS One. 2025 Sep 10;20(9):e0329296. doi: 10.1371/journal.pone.0329296 (PMC12422497; doi:10.1371/journal.pone.0329296)
Supplement: S1 Appendix — Data collection information and performance versus income graphs for all the states. (PDF) [file pone.0329296.s001.pdf]

Quantitative laws describing the dependence of K-12 student performance on the household income in U.S. school districts

Supplementary information

A Data sources and notes

Data on income were collected from the United States Census Bureau website, census.gov [1]. The census provides the American Community Survey (ACS) data collected in either 1-year or 5-year estimates. The 1-year estimates provide 12 months of collected data for areas with populations of $65,000+$, which is less reliable than 5-year estimates but contains the most current data. The 5-year estimates provide 60 months of collected data for all areas. While the 5-year estimate is not the most current, it is the most reliable as it allows for data for populations of all sizes. In this study, since we are seeking to compare income within Unified School Districts, we needed to ensure that we had data for all population sizes. We used the ACS 2019 5-year estimates for Median Household Income (table B19013) and the ACS 2019 5-year estimates for Per Capita Income (table B19301). The data for the 5-year estimates were collected between January 1, 2019, and December 31, 2023. The data on income reported is in 2019 inflation-adjusted dollars. The Census Reporter is an independent organization whose goal is to create a platform for others to use the information from the U.S. Census Bureau [2]. According to the Census Reporter, the Median Household Income (B19013) is the reported median income by household and not individual. Aggregate Income (table B19313) aggregates all income in the geography (such as school districts or cities). The Per Capita Income (table B19301) is the value for Aggregate Income divided by the total population for the respective geography. This means that the Per Capita Income is not the aggregate income divided by the number of people who have income but the aggregate income divided by the total population for the geography [2].

Data on state assessment scores were collected from each state's Department of Education websites. While many states publicly report their student assessment scores, some states were unable to provide an efficient way of obtaining the data to be analyzed. The following states were the ones

for which we were unable to obtain data for this research: Alaska, Hawaii, Maine, Michigan, Montana, South Dakota, Washington, and Wisconsin. We were able to collect, filter, and process the state assessment scores for the other 42 states for the academic year of 2018-2019. The table below provides a brief summary of each state's testing information as well as a link to the data source.

State	Link	Test information	notes
Alabama	[3]	Math and Reading	only has Reading and not ELA.
Alaska	[4]	ELA and Math	no excel file; must record district by district
Arizona	[5]	ELA and Math	Use 2019 AzMERIT assessment results
Arkansas	[6]	ELA and Math	
California	[7]	ELA and Math	
Colorado	[8]	ELA and Math	
Connecticut	[9]	ELA and Math	
Delaware	[10]	ELA and Math	
Florida	[11]	ELA and Math	
Georgia	[12]	ELA and Math	
Hawaii	[13]	ELA and Math	must generate data for each complex (what HI calls districts)
Idaho	[14]	ELA and Math	
Illinois	[15]	ELA and Math	Use ISAT results
Indiana	[16]	ELA and Math	Use ILEARN results
Iowa	[17]	ELA and Math	
Kansas	[18]	ELA and Math	Need to download Full Results once generate "State Totals" from selecting district
Kentucky	[19]	ELA and Math	
Louisiana	[20]	ELA and Math	
Maine	[21]	ELA and Math	unable to provide data requested
Maryland	[22]	ELA and Math	
Massachusetts	[23]	ELA and Math	
Michigan	[24]	ELA and Math	no excel file; must record district by district

Minnesota	[25]	ELA and Math	
Missouri	[26]	ELA and Math	
Montana	[27]	ELA and Math	no excel file; must record district by district
Nebraska	[28]	ELA and Math	
Nevada	[29]	ELA and Math	Generate report and download
New Hampshire	[30]	Reading and Math	
New Jersey	[31]	ELA and Math	
New Mexico	[32]	Reading and Math	
New York	[33]	ELA and Math	
North Carolina	[34]	Reading and Math	Sort by LEA for districts
North Dakota	[35]	Reading and Math	reported "range low" and "range high" of scores to suppress data information
Ohio	[36]	Reading and Math	
Oklahoma	[37]	ELA and Math	
Oregon	[38]	ELA and Math	
Pennsylvania	[39]	ELA and Math	Contact Educational Research Associate for data
Rhode Island	[40]	ELA and Math	Need to generate report with all districts
South Carolina	[41]	ELA and Math	
South Dakota	[42]	ELA and Math	Has data for each school within each district but does not have district totals
Tennessee	[43]	ELA and Math	Download District-level state assessment files
Texas	[44]	ELA and Math	
Utah	[45]	ELA and Math	Needed to contact Test Administration Specialist
Vermont	[46]	ELA and Math	Must download
Virginia	[47]	ELA and Math	Need to build a table with Divisions

Washington	[48]	ELA and Math	Data is too large to compile
West Virginia	[49]	Read and Math	
Wisconsin	[50]	ELA and Math	Difficulty in reporting Proficient and advance scores
Wyoming	[51]	ELA and Math	

B Fitting results for all the states

Figure S1 shows the results of ELA 3 performance versus median household income for the states where the nonlinear function (equation (1) of the main text) was the better fit. Figures S2 and S3 show results for the states where the linear function (equation (2) of the main text) was the better fit.

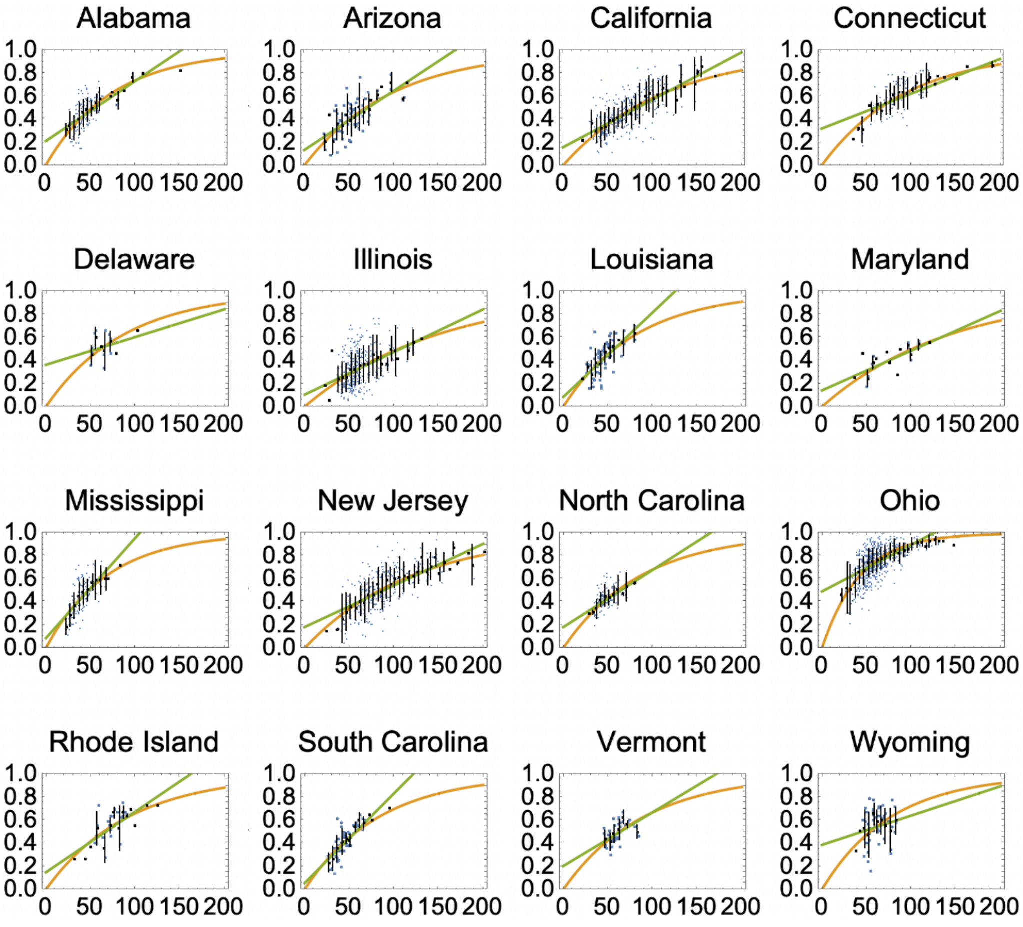

Figure S1: ELA 3 Performance score versus median household income for the states where the saturating function, $f_1(x) = 1 - e^{-ax}$, is a better fit. For notations please see figure 2 of the main text.

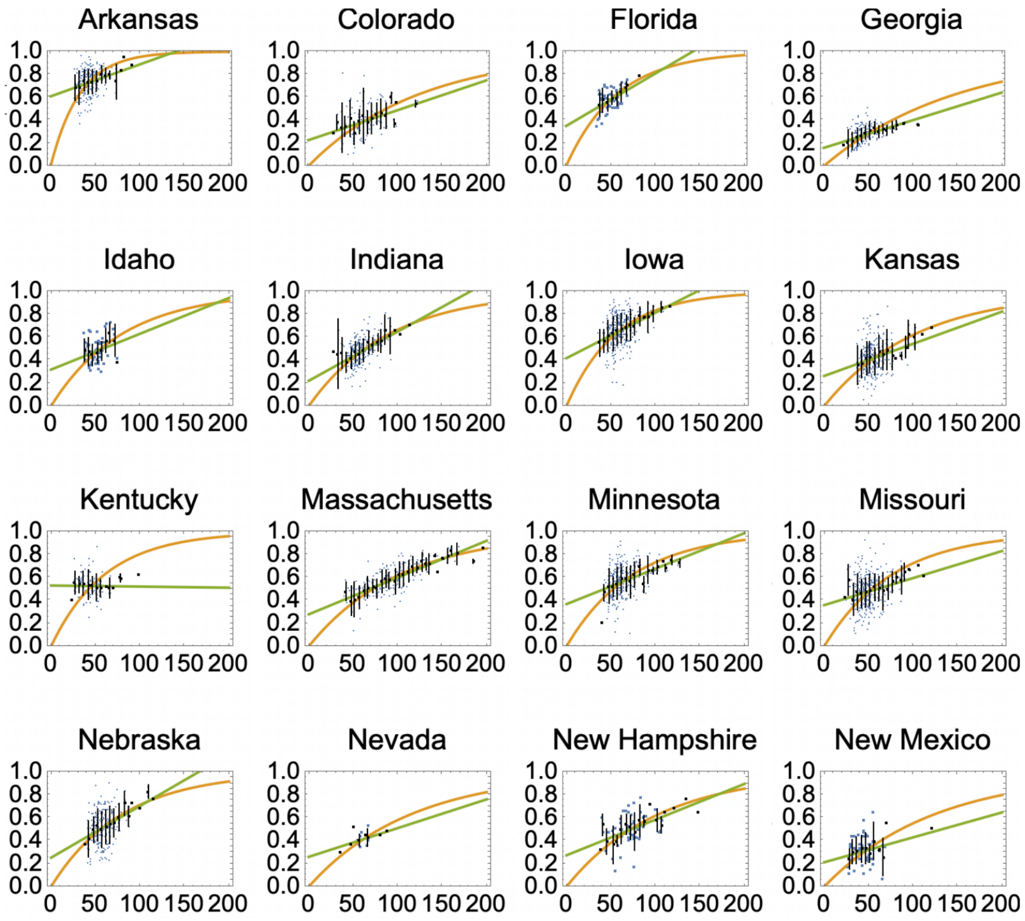

Figure S2: ELA 3 Performance score versus median household income for the states where the linear function, $f_2(x) = bx + c$ is a better fit. For notations please see figure 2 of the main text.

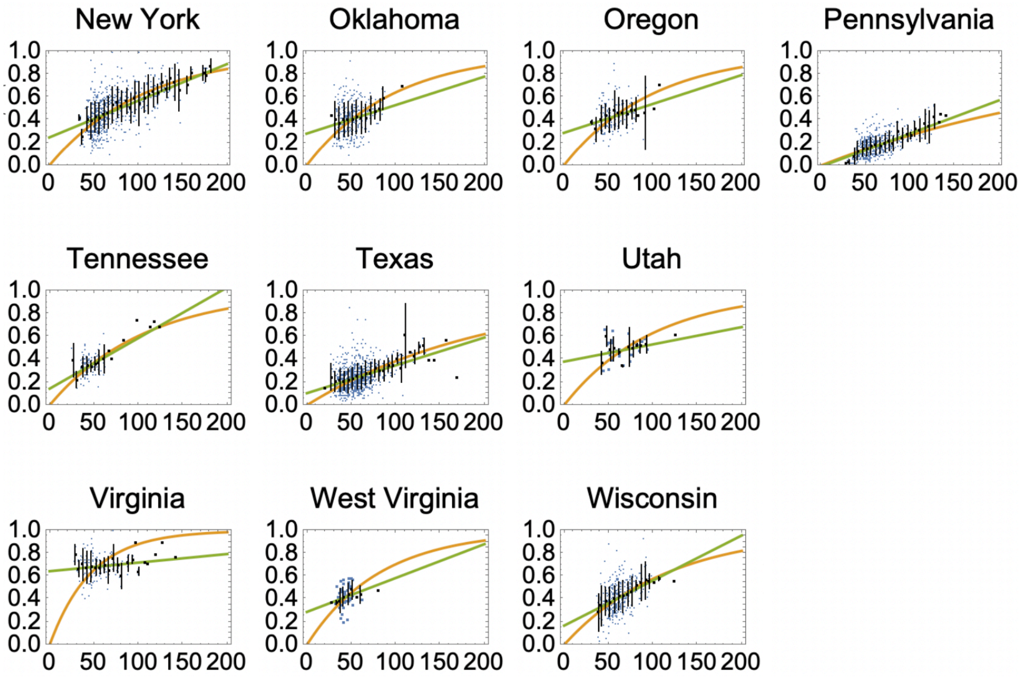

Figure S3: Continuation: ELA 3 performance score versus median household income for the states where function f_2 is a better fit. For notations please see figure 2 of the main text.

References

[1] United States Census Bureau. https://data.census.gov/. Accessed: September 2024.

[2] Census Reporter. https://censusreporter.org/topics/income/. Accessed: September 2024.

[3] Alabama State Department of Education. https://www.alabamaachieves.org/reports-data/school-performance/. Accessed: September 2024.

[4] Alaska Department of Education & Early Development. https://education.alaska.gov/assessments/results. Accessed: September 2024.

[5] Arizona Department of Education. https://www.azed.gov/accountability-research/data/. Accessed: September 2024.

[6] Arkansas Department of Education. https://dese.ade.arkansas.gov/Offices/public-school-accountability/assessment-test-scores. Accessed: September 2024.

[7] California Assessment of Student Performance and Progress. https://caaspp-elpac.ets.org/caaspp/ResearchFileListSB?ps=true&lstTestYear=2019&lstTestType=B&lstCounty=00&lstDistrict=00000&lstFocus=a. Accessed: September 2024.

[8] Colorado Department of Education. https://www.cde.state.co.us/assessment/cmas-dataandresults. Accessed: September 2024.

[9] EdSight. https://public-edsight.ct.gov/performance/smarter-balanced-achievement-participation?language=en_US. Accessed: September 2024.

[10] Delaware Department of Education. https://data.delaware.gov/Education/Student-Assessment-Performance/ms6b-mt82/about_data. Accessed: September 2024.

[11] Florida Department of Education. https://www.fldoe.org/accountability/assessments/k-12-student-assessment/results/2019.stml. Accessed: September 2024.

[12] Governor's office of student achievement. https://gosa.georgia.gov/dashboards-data-report-card/downloadable-data. Accessed: September 2024.

[13] State of Hawaii Department of Education. https://adc.hidoe.us/#/english-language-proficiency. Accessed: September 2024.

[14] Idaho Department of Education. https://www.sde.idaho.gov/assessment/accountability/. Accessed: September 2024.

[15] Illinois State Board of Education. https://www.isbe.net/Pages/Illinois-State-Report-Card-Data.aspx. Accessed: September 2024.

[16] Indiana Department of Education. https://www.in.gov/doe/it/data-center-and-reports/. Accessed: September 2024.

[17] Iowa Department of Education. https://educate.iowa.gov/pk-12/data/education-statistics#student-performance. Accessed: September 2024.

[18] Kansas Department of Education. https://ksreportcard.ksde.org/assessment_results.aspx?org_no=State&rptType=3. Accessed: September 2024.

[19] Kentucky Department of Education. https://www.education.ky.gov/Open-House/data/Pages/Historical-SRC-Datasets.aspx#InplviewHash502b1672-4397-43c5-aceb-047da39871be=FilterField1%3DYear-FilterValue1%3D2018%252D2019. Accessed: September 2024.

[20] Louisiana Department of Education. https://louisianabelieves.com/resources/library/elementary-and-middle-school-performance. Accessed: September 2024.

[21] Maine Department of Education. https://www.maine.gov/doe/Testing_Accountability/MECAS/NWEA. Accessed: September 2024.

[22] Maryland State Department of Education. https://reportcard.msde.maryland.gov/Graphs/#/DataDownloads/datadownload/3/17/6/99/XXXX/2024. Accessed: September 2024.

[23] Massachusetts Department of Education. https://profiles.doe.mass.edu/statereport/mcas.aspx. Accessed: September 2024.

[24] Michigan School Data. https://www.mischooldata.org/grades-3-8-state-testing-includes-psat-data-performance/. Accessed: September 2024.

[25] Minnesota Department of Education. https://public.education.mn.gov/MDEAnalytics/DataTopic.jsp?TOPICID=1. Accessed: September 2024.

[26] Department of Elementary and Secondary Education. https://apps.dese.mo.gov/MCDS/home.aspx?categoryid=2&view=2. Accessed: September 2024.

[27] Growth and Enhancement of Montana Students. https://gems.opi.mt.gov/student-data. Accessed: September 2024.

[28] Nebraska Department of Education. https://nep.education.ne.gov/#/data-downloads. Accessed: September 2024.

[29] Nevada Accountability Portal. https://nevadareportcard.nv.gov/di/main/assessment. Accessed: September 2024.

[30] New Hampshire Department of Education. https://www.education.nh.gov/who-we-are/division-of-educator-and-analytic-resources/bureau-of-education-statistics/assessment-data. Accessed: September 2024.

[31] New Jersey Department of Education. https://www.nj.gov/education/assessment/results/reports/. Accessed: September 2024.

[32] New Mexico Public Education Department. https://webnew.ped.state.nm.us/bureaus/accountability/data/. Accessed: September 2024.

[33] New York State Education Department. https://data.nysed.gov/downloads.php. Accessed: September 2024.

[34] North Carolina Department of Public Instruction. https://accrpt.tops.ncsu.edu/docs/disag_datasets/. Accessed: September 2024.

[35] Insights.nd.gov. https://insights.nd.gov/Data. Accessed: September 2024.

[36] Ohio School Report Cards. https://reportcard.education.ohio.gov/download. Accessed: September 2024.

[37] Oklahoma State Department of Education. https://sde.ok.gov/state-testing-resources. Accessed: September 2024.

[38] State of Oregon Department of Education. https://www.oregon.gov/ode/educator-resources/assessment/Pages/Assessment-Group-Reports.aspx. Accessed: September 2024.

[39] Pennsylvania Department of Education. https://www.pa.gov/en/agencies/education/data-and-reporting/assessment-reporting.html. Accessed: September 2024.

[40] Rhode Island Department of Education. https://www3.ride.ri.gov/ADP#. Accessed: September 2024.

[41] South Carolina Department of Education. https://ed.sc.gov/data/test-scores/state-assessments/sc-ready/. Accessed: September 2024.

[42] State of South Dakota Report Card. https://sdschoolreportcard.sd.gov/Studio/servlet/mstrWeb. Accessed: September 2024.

[43] Tennessee Department of Education. https://www.tn.gov/education/districts/federal-programs-and-oversight/data/data-downloads.html. Accessed: September 2024.

[44] Texas Education Agency. https://tea.texas.gov/student-assessment/student-assessment-results/staar-aggregate-data. Accessed: September 2024.

[45] Utah State Board of Education. https://www.schools.utah.gov/datastatistics/_datastatisticsfiles_/_reports_/_assessments_/RISEAspireProficiencyLevels2019.xlsx. Accessed: September 2024.

[46] Vermont Agency of Education. https://education.vermont.gov/document/vermont-education-dashboard-datasets-assessment. Accessed: September 2024.

[47] Virginia Department of Education. https://p1pe.doe.virginia.gov/apex_captcha/home.do?apexTypeId=306. Accessed: September 2024.

[48] Washington Office of Superintendent of Public Instruction. https://ospi.k12.wa.us/data-reporting/data-portal?title=&field_years1_target_id=1624&field_data_domain_target_id=All&field_level_of_aggregation_target_id=All&field_grade_span_target_id=All. Accessed: September 2024.

[49] West Virginia Department of Education. https://wvde.us/assessment/west-virginia-general-summative-assessment-3-8/. Accessed: September 2024.

[50] Wisconsin Department of Public Instruction. https://dpi.wi.gov/wisedash/download-files/type?field_wisedash_upload_type_value=Forward. Accessed: September 2024.

[51] Wyoming Department of Education. https://reporting.edu.wyo.gov/ibi_apps/run.bip?BIP_REQUEST_TYPE=BIP_RUN&BIP_folder=IBFS%253A%252FWFC%252FRepository%252FPublic%252FAssessment%252FDisaggregated%252F&BIP_item=AssessPerformDistrictHTML.htm. Accessed: September 2024.
